# Transformation Using Telepresence in the Classroom

**DOI:** 10.1007/s10606-026-09542-8

**Published:** 2026-06-03

**Authors:** Jennifer A. Rode, Madeline H. Samson, Hanlin Zhang, Yifan Feng, Adam Walker, Xinyue Dong, Martin Oliver

**Affiliations:** UCL Knowledge Lab, University College London, London, UK

**Keywords:** Telepresence robots, University, Theory, Ethnography, Courtesy, Embodied interaction, Articulation work

## Abstract

This paper explores best practices for computer-mediated communication through mobile telepresence robots in educational settings. We present findings from a longitudinal ethnographic study of a hybrid class where students and professors used telepresence robots to participate. We discuss the challenges of articulation work in telepresence robots, particularly around being perceived as courteous. We discuss Murray’s theory of transformation as a goal of telepresence to ensure remote students feel fully included in the classroom. We argue with regards to telepresence that when transformation occurs, you feel fully a part of the remote environment. Our contribution is the use of Grounded Theory (GT) to argue that prior research on articulation and courtesy work can be extended to explain how users overcome challenges that impede transformation. When transformation does occur, telepresence affords deeper embodied participation than possible with traditional videoconferencing applications.

## Introduction

**Fig. 1:**
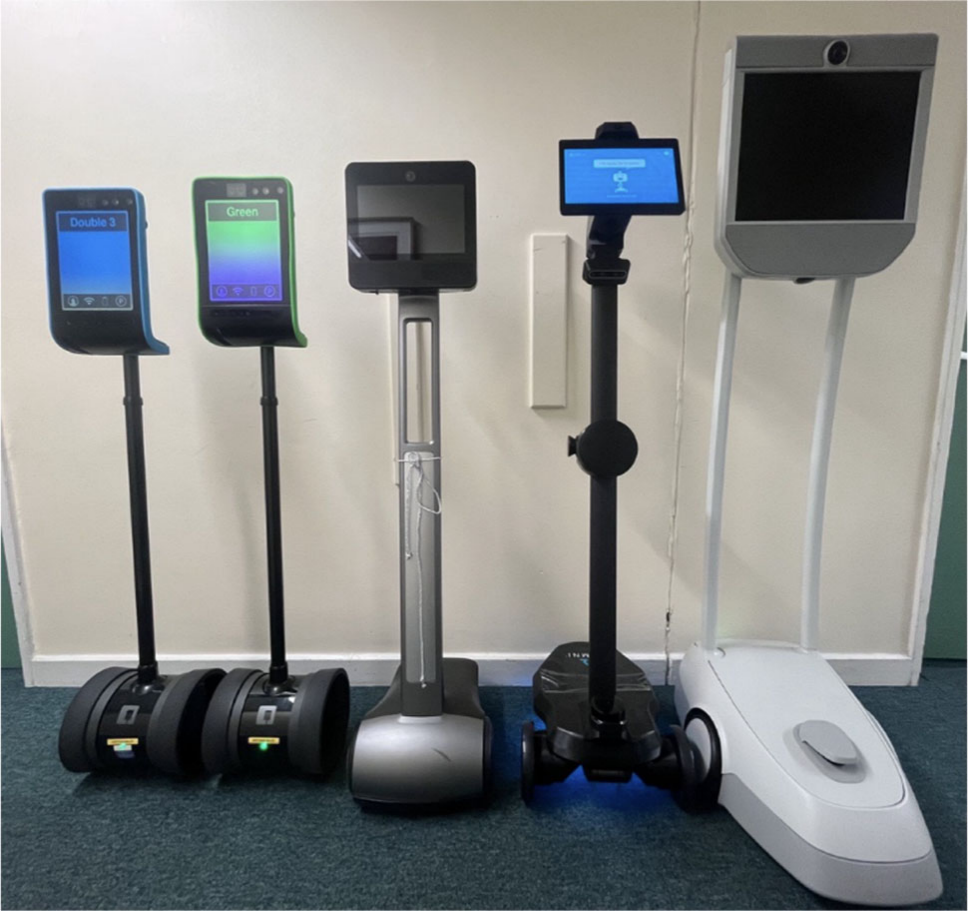
Our study’s telepresence robots. From left are two Double 3 robots, Beam+, Ohmni Pro, and Beam Pro

Robotic telepresence technologies play a unique role in computer-supported collaborative practices. Recent Eusset Conference on Computer-Supported Cooperative Work (ECSCW) scholarship has focused on robot interactions and use in community and public spaces (Joshi 2019; Joshi and Sabanovic 2019), office (Abe and Colombino 2024), home (Ciolfi et al. 2020), and health and elderly care facilities (Unbehaun et al. 2019; Nauwerck and Cajander 2019). Johansen et al. (2024, p. 2) argue that this type of technology has more*‘embodied agency’* compared to other technologies typically investigated for collaborative interaction in the field and suggest further research into the materiality of a diverse array of robots and their applications to understand better how they mediate communication and facilitate collocated social practices, and yet, deep ethnographic studies of embodiment in a university classroom context do not exist. Here, we present a term-length ethnographic study of graduate students with three brands of mobile telepresence robots (MTRs) (see Fig. 1) for remote students. Unlike other studies of telepresence robots, students did not use the robot for a single session, but had sustained organic engagement motivated by their own unique educational needs. Our open-ended research question centered around exploring how students use telepresence robots to mediate their participation in classroom settings when they are physically remote and how they accomplished this work to become part of the class.

In discussing our findings, we take up a central issue in Human-Computer Interaction (HCI) and Computer-Supported Cooperative Work (CSCW) concerning computer-mediated communication (CMC) and authentically conveying one’s identity when participation is mediated through technology. Our empirical contribution is that we discuss CMC with regard to telepresence in a classroom setting for the first time. Identity presentation has been considered extensively in work on teleworking (Olson and Olson 2000; Christensen 1987; Kirk et al. 2010). As we engaged in abductive coding, Janet Murray’s (Murray 1998) theory of transformation was identified as central in understanding these interactions. Murray (1998) explores how a user can feel fully part of a remote environment through a process she calls “transformation.” In this paper, we use our ethnographic findings to show this theory extends to telepresence. Related to this, it is the student’s embodied experience (Dourish 2001) that allows the student to achieve transformation, which is critical to remote students feeling like they are truly part of a hybrid classroom. We position Murray (1998)’s “transformation” as a goal of telepresence robot use. We examine how articulation work and what we call “courtesy work,” the work to be perceived as polite by your classmates and teachers, are critical achieving transformation. By extending Murray’s theory of “transformation” to telepresence, we discuss the point where the division between self and robot disappears, and one has agency and is fully immersed in the physical world using a robot-mediated body.

The following ethnographic vignette to illustrates the relevance of Murray’s transformation framing the paper. The following vignette illustrates a moment where transformation arose for the students:

It never occurred to me that you could play keep away with a telepresence robot. Thus, it was to my exceptional surprise after class when I looked up and saw Nandu and RA1 playing and laughing in a good-spirited fashion while chasing each other across the classroom. Nandu was attending via Beam+ robot calling in from India, and RA1 had been providing him classroom-based technical support. Every time one of them approached the other, they would dart off in a different direction laughing happily. Even as an anthropologist, this sort of technologically mediated interaction was something I had never considered. Both of these young men were graduate students... there was something magical about this technology that afforded grown men childlike joy. In this moment, Nandu had achieved a fully embodied experience, having both immersion and agency and achieved what Murray called transformation...despite being half a world away, he was also in my classroom playing with his dear friend. (FN-Prof-W1)

We argue that transformation, in this sense, is the goal of telepresence in the classroom. Throughout this paper, we will explore both the challenges inherent in achieving this transformation and the compelling reasons that make it a worthwhile pursuit, even in the face of technological shortcomings.

The aim of this paper is to develop and extend existing theories, connecting the analysis of our ethnographic findings using Grounded Theory to Murray’s theory of transformation to telepresence. Just as Murray argues transformation was critical to virtual reality mediated interactions, we show it is similarly relevant to telepresence use and demonstrate how articulation and courtesy work are deployed by students in response to barriers to achieving tranformation. We argue that when transformation occurs, telepresence enables a deeper embodied participation than is possible with traditional videoconferencing applications. This underscores transformation as a critical element of embodied interaction, an element notably absent from other theories of CMC.

## Related Work

### Telepresence in Higher Education and Beyond

The pandemic highlighted the limitations of traditional remote meeting software, particularly the lack of meaningful presence (Winter et al. 2021), which compounded challenges for isolated individuals (Banks et al. 2001). Research shows students prefer in-person to online class attendance (Paechter and Maier 2010; Meda and Waghid 2022) and that disability is a major cause for missing school (Disability Rights UK 2023; Banks et al. 2001). With 11% of children in the UK (Department for Work & Pensions 2022) and 11% of our university student body identifying as disabled, telepresence offers a promising solution to keep these students connected to education. Telepresence robots, featuring videoconferencing and locomotion, allow students to physically participate in classes and social events from remote locations via a robotic body (Leoste et al. 2022; Reis et al. 2018). Several studies on telepresence robots and hybrid education have focused on learners’ experiences with commercially mature robots, including stationary robots, such as the AV1 (Neumann et al. 2025, as well as semi-static tablet-based robotic systems, like the Kubi robot (Cain et al. 2016; Bell et al. 2016; Hei et al. 2023; Arguga and Kashihara 2024) and the Fable Connect robot (Weibel et al. 2024). Among these studies, students are found to report a greater sense of physical presence and learning and social engagement compared to traditional software-based videoconferencing systems (Arguga and Kashihara 2024); however, this experience is less positive compared to mobile telepresence (Hei et al. 2023. Recent work on mobile telepresence further explored areas such as utility and usability in supporting distance learning (Hu et al. 2024; Lister et al. 2018; Shaw et al. 2018), (co)-presence and interaction (Fitter et al. 2020a; Lei et al. 2019; De Jong 2021; Fitter et al. 2020b; Elmimouni and Sabanovic 2025), perception and acceptance (Lei et al. 2022), shared learning environments in online/hybrid education (Capello et al. 2024; Gallon et al. 2019; Thompson and Chaivisit 2021; Jakonen and Jauni 2022; Berhane et al. 2025), and instructional design and teaching practices (Corsby and Bryant 2020; Jakonen and Jauni 2021; Rinfret 2020), particularly in relation to its improved mobility affordances. Some work has also occurred in office settings, creating technical improvements to address the “uncanny valley” and the lack of spatial awareness due to the absence of visual cues from robotic body positions (Jones et al. 2021). These studies particularly examined telepresence’s affordances and functionalities, providing insights into the feasibility and potential of adopting telepresence in equitable and accessible learning/work environments. In contrast, Elmimouni et al. (2024) investigated user values with respect to the physical form and presence of telepresence in classrooms. They highlighted three core user values, namely identity, privacy, and courtesy, that played an (inter)active role in shaping co-located social interactions and classroom dynamics. To our knowledge, this is the only study extensively researching human values in relation to educational uses of telepresence, to date. Exploring the value tension between user and design values is a core element of Value Sensitive Design (Borning and Muller 2012) and a key reason classrooms in particular need to be studied to unpack this value tension, as classrooms with their focus on education and personal growth have different social and economic goals from offices and technological developments.

Given how little directly relevant prior work exists, we also considered other ethnographies of telepresence. For example, Thach et al. (2023) focused primarily on video calling instead of telepresence robots and looked at the benefit of video-calling devices for people in residential-aged care. Thach et al. (2023) found that there were social and emotional benefits for residents, as they could communicate with loved ones via telepresence technology. However, there were challenges sustaining this use of technology throughout the study due to staff workload and cultural values related to technology use. The autoethnographic ethnomethodological study of Boudouraki et al. (2022) supports this, suggesting that MTRs allow remote users to feel physically present when visiting others’ homes. This is because they allow users to move around the environment they are visiting independently, providing more spontaneity in social interactions than would be observed in in-person visits. However, while these studies provide rich data on the nature of people’s mediated communication using telepresence, neither occurred in a classroom context. Also, while both provide rich qualitative data in a non-school context, neither aimed to generate HCI theory addressing interaction.

One critical theory of interaction is Goffman’s discussion of identity. Goffman explains that one’s identity is made up of various characteristics, including one’s appearance, attitudes towards others, beliefs, and emotions, and that it is presented to others through interactions and actions (Goffman 1949). Goffman’s identity is particularly relevant in this context as it helps analyze how telepresence users negotiate their social identity through robotic representations. In research about telepresence robots, Elmimouni et al. (2023) found that human-height screen displays and movement support identity projection; however, challenges such as spatial and audio ambiguity, with the dominance of robot identity, can undermine users’ self-perception and individuality. Therefore, given that the use of telepresence involves more than just technological interaction, further study is needed to explore values, robotic affordances, and user experiences, enabling a more inclusive and empathetic approach to integrating such technologies into Higher Education.

### Articulation Work and Telepresence

Articulation work is a core theoretical concept throughout HCI and CSCW (Gerson and Star 1986; Schmidt and Bannon 1992; Suchman 1996; Star and Strauss 1999; Grinter 1996; Strauss 1988). It stems from Strauss (1988, p. 24) who defines it as “the specifics of putting together tasks, task sequences, task clusters — even aligning larger units, such as lines of work and sub-projects in the service of workflow.” Through a feminist lens (Suchman 1996), subsequent research engaged with its political nature, highlighting less visible work and meta-labor necessary for designing and using labor-sensitive sociotechnical systems (Star and Strauss 1999; Sawyer and Tapia 2006; Dew et al. 2019). Consequently, this implies that articulation work is inherently context-dependent and primarily relates to labor and technology practice in professional work environments. However, these experiences may vary between workplace and school settings. For example, Stevens (2000) observes distinct differences in labor divisions between schools and design firms when completing collaborative design tasks. His findings suggest that the unique norms and power dynamics of each setting, along with an understanding of the social and material contributions of teams and individuals, shape professional designers’ and students’ choices about using either computer-based tools or paper-based tools to achieve collective goals in different stages of design. This indicates a need to design and evaluate technology functionalities and capabilities in light of context-dependent practices and social-cultural norms and values. Overall, while articulation work is well-established in HCI, particularly concerning office-based telepresence use, there remains a need to evaluate this when researching MTR’s applicability in a classroom context.

Recently, Rode (2018) and Elmimouni et al. (2024) have extended articulation work to telepresence in educational settings. Rode (2018) provides examples of the articulation work needed to manage a telepresence robot, such as keeping tabs on battery life and WiFi signal, ensuring visibility and audibility, and handling the telepresence-mediated sense of touch. For example, Rode describes the palpable panic of managing battery levels to avoid logging out mid-conversation. Elmimouni et al. (2024) highlight how the users’ values around identity, privacy, and courtesy are key motivators for articulation work. However, Rode (2018)’s work is auto-ethnographic, and occurred largely in conference settings, and Elmimouni et al. (2024)’s study focused on students’ participation in a single session. What we have not seen are studies following students in real classes for extended periods of time. Thus, studying classrooms “in the wild,” longitudinally, where students’ real grades and learning experiences are on the line, remains an important unstudied area for articulation work.

### Performing Courtesy in the Classroom

One key kind of articulation work that we witness was trying to follow the complex social codes of courtesy and appropriate behavior to which students are expected to adhere (Kern and Clemens 2007) in the classroom. In educational settings, courtesy plays an integral role in fostering positive interactions among students, educators, and staff (Haslip 2020; Santamaría-García 2017; Mahmud 2019) and creating spaces conducive to learning (Mahmud 2019). With this in mind, numerous studies exist to understand and evaluate the concept of courtesy within the primary, secondary (Lo 2009; Howard 2009) and post-secondary (Santamaría-García 2017; Mahmud 2019) education. However, how politeness is performed in a university setting with telepresence robots is largely unexplored, save for Elmimouni et al. (2024).

Politeness is culturally constructed and situated through social norms, with significant cultural differences between the East and West (Yang 2013; Hall 1976). Brown and Levinson (1987) in their seminal, but admittedly Western-focused work, discuss a central role of politeness as an individual’s navigation of social interactions to maintain ‘face,’ or one’s desired self-image. Positive face refers to the inherent desire of every individual to be liked, appreciated, and understood by others; conversely, negative face refers to the desire of every individual to act freely without interference from others (Brown and Levinson 1987). Brown and Levinson (1987) argue the central purpose of being courteous is to protect one’s own face, as well as the faces of others. They propose various politeness strategies to achieve this, such as demonstrating interest and sympathy, using in-group identity markers, seeking agreement and avoiding disagreement, asserting common ground, and being optimistic.

Although Lee et al. (2011) examined how remote workers engage in informal communication via telepresence robots and develop social connections by negotiating new forms of courtesy, what this means for users of telepresence robots in classroom contexts is largely an open question. Elmimouni et al. (2024) highlight apologies as a way to compensate for surprising someone by being able to manipulate a robot remotely. Conversely, they describe the discourtesies of referring to a human driver as a robot, recommending a new social norm of highlighting the humanity of the human driving the robot. While Elmimouni et al. (2024) highlight the need to study courtesy practices, a thorough explanation of the emergent courtesy practices while using robots remains under-explored. One key aspect of courtesy centers around managing one’s remote robot body, which we will turn to next.

### Embodiment, Immersion, Agency, and Transformation

Telepresence robots are unique due to their material embodiment. Dolezal (2009) uses a phenomenological lens to re-examine the embodiment afforded by telepresence systems. Björnfot (2022) builds upon this by proposing a refined concept, “remote embodiment,” that describes the mediating effect of robots on users’ perceptions and proprioceptive feedback while facilitating their connection to the physical world. Particularly, Takayama (2015) pointed out how telepresence robots’ physical form affords apparent agency for remote users to feel and be treated as co-located with local participants. This form also shapes the dynamic impression management of local participants to overcome distance barriers for effective collaboration (Kornfield et al. 2021). Through design exploration, Kaerlein (2012) highlights that this bodily integration for tele-mediated communication can “evoke a feeling of presence” and provide more direct and intermediate communicative exchanges, leading to increased emotional attachment to places (Hu et al. 2025) and a sense of social inclusion in educational settings (Elmimouni and Sabanovic 2025). However, Boudouraki and colleagues (Boudouraki et al. 2023b; a) conducted ethnomethodological research in a global technology company and found that telepresence robots were underutilized. Workers perceived them as offering limited added value, particularly in an environment already well-supported by existing hybrid work infrastructures, and regarded the robots as poorly aligned with the demands of distributed workflows. The authors further theorize that, despite adherence to politeness norms, telepresence robots were frequently “othered” and failed to attain the social integration necessary to be perceived as “ordinary.” These studies illustrate that embodiment is a well-researched area of telepresence, although it has not been discussed in a classroom context.

In contrast, Rode (2018) took a different approach to material embodiment longitudinally through her lived experience as a disabled researcher using telepresence. Rode writes, “Telepresence robots are distinctive in the way that they remediate the experiences of corporeal bodies, allowing the user to experience and participate in remote environments.” You have a corporeal body and a technologically mediated presence elsewhere. This distributed assemblage will allow the user to feel connected to their remote environment. We foreground the work of Rode here, as this ethnographic study builds on Rode’s own prior auto-ethnographic research. As such it is critical to understand her prior work to understand her positionality and lived experience with respect to telepresence mediated embodiment.

While Dourish’s theory of embodiment well predates robotic telepresence, it proves especially relevant when bridging our corporal body with an embodied physical presence in another space. Dourish (2001)’s use of the concept of embodied interaction does not refer purely to the physical but rather the participative status of everyday life. Dourish discusses embodied interaction as the site where humans interact with computer systems which “occupy our world, a world of physical and social reality, and that exploit this fact in how they interact with us,” (Dourish, 2001, p.3) and they do so to perform mundane tasks. Thus, (Dourish, 2001, p.126)’s embodied interaction is “the creation, manipulation, and sharing of meaning through engaged interaction with artifacts.” In this article, we will extend the relevance of this theory to mediated interactions with the mundanity of the classroom through the mobile telepresence robot.

While Dourish focused on ubiquitous computing, Murray (1998) focused on participation mediated through virtual worlds, largely through virtual reality. She focuses on three ways in which the user can feel a sense of embodied interaction with their virtual environment. First, Murray describes immersion as the “experience of being transported to an elaborately simulated place” (Murray, 1998, p.98). Later, she defines immersion as,

a metaphorical term derived from the physical experience of being submerged in water. We seek the same feeling from a psychological immersive experience that we do from a plunge in the ocean or swimming pool: the sensation of being surrounded by a completely other reality, as the sensation of being surrounded by a completely other reality, as different as water from air, that takes over all of our attention, our whole perceptual apparatus (Murray, 1998, p.98).

Murray was concerned with participating in virtual worlds, often as part of narrative video games. Here, we extend her theory to telepresence. For a telepresence user, this means, do you feel like you are in your dorm room at an interface, or really in the classroom with everyone else? Second, within this theory, Murray defines agency as “the satisfying power to take meaningful actions and see the results of our decisions and choices” (Murray, 1998, p.126). In a telepresence context, if you can push a chair across the classroom or make contact with a classmate intentionally, you have agency, whereas if you have to ask someone else to move a laptop cable out of the way, press an elevator button, or open a door, your agency is relatively reduced.

To clarify, we engaged with Murray’s understanding of agency to investigate telepresence-mediated social practices rather than Giddens (1979)’s agency, as discussed in prior HCI & IS literature (Jones and Karsten 2008; Gibbs et al. 2021; Räsänen and Nyce 2006; Kuutti and Bannon 2014). In part, this is a practical decision, in that Murray’s concept of transformation builds on her own framing of agency. Additionally, while Giddens’ framework assists technologists in understanding how users’ interactions with technology shape and are shaped by their technological and social contexts, Murray emphasizes the “satisfying power” that enables users to take “meaningful action” within a digital environment. Unlike Giddens, who focuses on interaction, Murray concentrates more on interactivity—a feeling of participation driven by meaning. This perspective aligns with Dourish’s discussions on how meaning is communicated through embodied interactions.

Finally, Murray introduces transformation as a desirable endpoint of VR interactions dependent on having achieved both immersion and agency. She defines transformation in a VR game context as the “power of the computer... it makes us eager to masquerade, eager to pick up the joystick and become a cowboy or a space fighter, eager to login... and become ElfGirl or BlackDagger.” In this paper, we will use the term ‘transformation’ for situations where the telepresence user’s experience is immersive and gives rise to agency, such that they feel like they have become someone else; they are no longer “a distance learner” or home sick, but instead have become part of the class that meets in a specific room at a specific university in a specific city, which could be half a world away. This paper will explore the relationship between embodied interaction and transformation in the context of telepresence robots.

Extending these discussions of embodied interaction and transformation, Vertesi (2015) explores how robots’ drivers experience the world without being aware of the mediating role of the robot. Vertesi builds on Dourish’s discussion of Heidegger (Dourish 2001), particularly the idea of technology being “ready- or present-at-hand”. Technology is “ready-to-hand” when used without conscious effort - for instance, just now the wireless keyboard is an extension of my fingers. However, technology is present-at-hand when we are consciously aware of it, such as in moments of breakdown, for example if my keyboard runs out of battery. Thus, Vertisi’s Mars rover robot is mostly ready-to-hand, with the user’s focus being on the meaningful task, not the interaction with the interface that controls it. Building on this distinction, Rode (2018) discusses what she calls “handless feeling”: the notion that her corporeal body feels the remote environment, as illustrated by this vignette:

When I hit something, intentional or otherwise, the bottom of the robot stops abruptly, but the camera seated at the top of the robot, my portal to the world, continues forward until the center of gravity jerks it back. When this jostling of the camera occurs unintentionally and my attention is wholly immersed in my virtual presence, I find myself jerking my head back as the camera goes far too close to a door or other object and the hairs on my arms bristle. It is not painful, but the experience of hitting something remotely is nonetheless embodied.

This illustrates Dourish’s proposal that designing for embodied interaction should avoid the Cartesian mistake of assuming a dualism between mind and body. Murray’s theory of transformation and Dourish’s use of Heidegger have not to our knowledge been considered in light of each other before. We argue that when immersion, agency and transformation occur, the technology is “ready-to-hand,” and when they fail they are reduced to being merely “present-at-hand.” In this paper, we explore how embodied interaction affords transformation.

## Method

Our ethnographic data was collected in the first author’s class as part of a small masters-level hybrid class on ethnographic methods at the University College London. The study occurred over three months in the Spring of 2024, which included ten weekly sessions, a mid-term break week, and a week with final presentations. There were two weeks during which the robots were not used: the mid-term break week and the term’s last week due to personal obligations. Throughout the paper, we refer to weeks 1-10 as the ten weeks participants used the robots. We present detailed information on robotic designs and their use, student attendance, and other relevant information in the following sections.

Students had the option to take part in person, or by using MTRs, Zoom, or both, as part of a hybrid class. Our study catalogs the practices of individuals who elected to use the robots week in and week out and a number of students who elected to use the robot for a shorter duration (often to give them an increased presence in the classroom during an illness).

The class taught ethnographic methods in HCI and Education, so students were learning how to take field notes and do data analysis, while also participating in an ethnography, making many of the examples in this paper very meta. However, this context also explains their eagerness to participate, as they saw value in both using the technology and seeing first hand how an ethnography was conducted. The first author, an anthropologist, was the instructor of this class. Students elected to attend class sessions in person, via telepresence robot, or through Zoom, depending on their preferences and changing circumstances. However, many gravitated towards the telepresence robots, finding them novel and generally perceiving them as a superior A/V setup for participating in the class remotely compared to traditional video conferencing with Zoom, which relied on desktop and room-based cameras, microphones, and screens. Participation in the study was entirely optional, did not impact grades, and was approved by the university’s ethics board (#REC1766). With this in mind, students could drop out of the study or switch to Zoom-only participation at any time. Additionally, they decided whether to be referred to by pseudonyms in the findings or to be credited directly, with all preferences fully respected.

### Confessional Ethnography

The study employed anthropological ethnography as its research method (Rode 2011), and as such we engaged in participant observation and used this evidence to develop and extend theory. Clifford (1996) refers to a discussion with Rosaldo where she described participant observation as “deep hanging out” when one takes on a formal role relative to the participants. Later, Salvador et al. (1999) argued the importance of this approach to design in computing. Elsewhere, Bell (2001, p. 1) writes,

This means that most anthropologists (and those from other disciplines who use ethnographic methods) do field work. They spend time in and with the cultures and peoples they are studying, engaging with the people around them, participating in every-day life, and attempting to make sense of the patterns of that culture.

Thus, having the first author serve as the instructor of the class aligned with best practices for participant observation. Similarly, other team members assumed roles such as teaching assistant (TA), guest lecturer, or tech support to allow appropriate and seamless access to the classroom. This approach allowed students to view these individuals not as external observers, but as active contributors to the class– teaching, facilitating technology, and incidentally taking notes as part of their roles. In writing up our data, we followed the established conventions for ethnographic research. While realist ethnographies are likely more familiar to the broader HCI & CSCW audience given their greater adherence to positivist traditions (Brown et al. 2007; Randall et al. 2021; Blomberg and Karasti 2013; Randall et al. 2005), confessional ethnography is better established within anthropology (Van Maanen 2011). Confessional ethnography intentionally uses “reactivity” (Burawoy 1998), allowing the ethnographer to discuss their relationship with their informants and how they gained rapport (Rode 2011; Van Maanen 2011). This method has been used in HCI by anthropologists such as Bell (2006) and Rode (2011). Given the clear power dynamic between a professor studying their own students, a realist framing that did not question the influence of this relationship would have been ethically problematic. In a confessional ethnography, “Stories of infiltration, fables of fieldwork rapport, minimelodramas of hardships endured (and overcome), and accounts of what the fieldwork did to the fieldworker are prominent features” (Van Maanen, 2011, p. 73). To achieve a confessional tone, we followed ethnographic conventions of “thick description” (Geertz 1973), because as Rode argues, “[e]xplaining the strength of rapport to the reader goes beyond the spoken word, to include the wink, the innuendo, the shared glance, the comportment of the body, and tone.” (Rode 2011). Thus, instead of simply including participant quotes throughout the paper, we present extremely detailed ethnographic vignettes from our field notes that both present the data and reflect on the researcher’s relationship with the participants.

As in other anthropological confessional ethnographic texts, data and interpretation cannot be separated. Thus, here, we discuss them together, following this ethnographic convention. This is consistent with our use of Constructivist Grounded Theory (C-GT) as a methodology (Charmaz 2006): As themes are co-constructed by the researcher and participants, it is difficult (and can be inappropriate) to neatly separate the processes of presenting, interpreting and discussing evidence. The structure of this paper reflects this, reporting evidence (itself an interpretation of experience) and discussing it so that inferences are closely linked to the ’thick descriptions’ of practice that provide a warrant for them. As the contribution of this work is the development of existing theories to explain classroom experiences, and because our work is reflexive, we did not seek to generalize our findings to other settings where telepresence is used, nor did we create implications for design. Instead, our development of theory shows that these ideas can be extended to different settings, but like any theory-building work, this involves careful working through of the appropriateness of these ideas to the new context.

### Participants

In total, 12 mixed-ability students (5 female, 7 male) enrolled in the class as part of their degree requirement; 10 students (5 male, 5 female) agreed to participate in the study. Five participants had non-visible disabilities such as neurodivergence, mental health conditions, and diabetes. To ensure participants’ privacy and anonymity and respect their choices, particularly given the small sample, we have opted to avoid including additional demographic information^1^. Though this decision supports confidentiality, it also means certain demographic nuances (e.g., exact age or overlapping disabilities) are not fully detailed, limiting the granularity of our analyses. However, we outline participants’ telepresence use and duration and students’ attendance in Table 1 and Appendix C.

Our ethics board approved the use of telepresence robots in the classroom with students who declined to participate in the study, provided no data was collected on them– a condition we strictly adhered to.

The class included an international cohort. Among the study participants, 6 students registered as face-to-face students (4 female, 2 male). Of these, 3 occasionally used robots when ill (Xiaoke-F, Olive-F, and Jesse-M), while the remaining 3 (Jo, David and Dan) attended entirely in person.

3 students were distance learners, participating remotely, and all used robots (Richard-M, Cyrus-M and Tasha-F). Both male students drove the robots consistently throughout the term, while the female student drove only once. Another student (Nandu-M) drove the robot for the first two weeks while abroad before transitioning to face-to-face attendance. Distance learners participated from locations including India, Thailand, United Arab Emirates, and the United Kingdom.

Two faculty members also used robots during our study. In Week 3, a professor on the research team attended via robot, and the first author who was the class’s primary professor, attended via robot during Weeks 9 & 10 while recovering from surgery.

A breakdown of attendance and which robots were used is provided in Table 1. The technical specifications of each robot are provided in the following section (Section 3.3).

**Table 1:** Robot use by week. Includes type of participant (indicated by first three letter of their pseudonym) followed by type of robot. Participant types: DL=Distance Learner, Ill=Student who was sick that week and participated remotely, Prof=Professor. Robot types: B+=Beam+, D=Double, BP= BeamPro, O=Ohmni. *=Connection lasted 5 minutes before battery died. ()tried additional robot

Week	Nan	Ric	Tas	Xia	Cyr	Oli	Jes	Prof	VProf
1	DL-B+	DL-D	DL-D						
2	DL-B+	DL-D		Ill-BP	DL-D				
3					DL-D				Prof-B+
4					DL-D	Ill-D			
5		DL-D					Ill-D		
6		DL-BP							
7		DL-BP							
8				Ill-BP	DL-O				
9					DL-O*			Prof-B+	
10		DL-B+ (D)						Prof-BP	

### Telepresence Robots

Most studies of telepresence robots use a single vendor’s robot. While we had initially envisioned using three GoBe robots to support the class, we could not maintain stable connections with them. Instead, we used a mixture of robots borrowed from colleagues, which conveniently mirrored the reality of bring your own device (BYOD) policies for laptops in classrooms where you typically have to support a range of vendors (Davis and Kohun 2018). We had five robots in the classroom including two Double 3, a Beam Pro, a Beam+, and an Ohmni Pro robot, which arrived during Week 8 (Table 2).

**Table 2:** Description of telepresence robots’ specs

Feature/Robot	Double 3	Beam Pro	Beam+	Ohmni Pro
Height	120–150 cm	158.7 cm	134.4 cm	144.2 cm
Adjustable Height?	Yes	No	No	No
Navigation Approach	Arrow key, click screen	Arrow key, click & hold, game-pad controls	Arrow key, click & hold, game-pad controls	Arrow key, option to use mouse to rotate
Collision Detection	Yes (cannot be deactivated)	Yes	No	Yes (can be deactivated)
Other Features	Adjustable height	–	–	Tilting head

These robots all had the same core features allowing the remote user to move about campus and zoom in. However, they all had slightly different additional features. The screens of both Beam robots (Beam+ and Beam Pro) were static, whereas the Doubles were height adjustable, and the Ohmni Pro had the ability to pivot the display up and down. The Beam Pro was the same scale as a human, whereas the other four robots projected their users as smaller than human size. All robots had the ability to display small bits of text on screen; this was commonly used for the user’s name. Only the Double 3 had the ability to display websites.

Robots also differed in whether they had collision detection - in other words, if you could physically make contact with the classroom environment. This could be done accidentally, e.g. by knocking a classmate’s chair, or intentionally, as seen by a student who tried to play tag with his classmates. Collision detection being engaged would have the side-effect of limiting the robot’s ability to be driven through narrow spaces, like the aisles between rows of chairs in the classroom. The Double 3 robots had collision detection, but this also meant that users could navigate by just clicking where you wanted to go and letting the AI plot a course. The other robots did not have AI assistance. The Beam Pro had collision detection permanently turned on. The Beam+ and Ohmni Pro had collision detection that could be toggled on and off; we let users decide whether to use it.

Students were able to select which robot they wanted to use each week. The student who reached out first each week had first pick of the available robots. This was typically the distance learners who used the robots as the primary means of attending. Students who were frequent users of the robots tried multiple robots, with and without collision detection. Students who were sick, especially those who learned they had COVID, reached out at the last minute and were content with whichever robots were still available. Ultimately, we had more robots than students who wanted to use them, and everyone was content with their access to robots.

### Data Collection

In total, 46 sets of ethnographic field notes were taken that ran over 291 pages of thick description (Geertz 1973). This included field notes by the professor and TA, who took jottings during the class, and wrote ethnographic field notes afterwards. We had three research assistants, two of which were MA students on the same course as this class. Each week, two RAs attended the class, with one providing technical support and one focusing on taking field notes. In practice, sometimes they both provided technical support. One student, Olive, took auto-ethnographic field notes on her own participation in the class, as she was also using this data as basis of her own dissertation. Finally, another senior faculty member visited some classes (including via robot in Week 3) and took additional field notes, focusing on educational pedagogy. In total across the study, we had seven people taking ethnographic field notes, although most weeks only 4 individuals took notes. Thus, even while the professor and TA had split focus between jottings and teaching, and Olive had split focus on her own coursework and her auto-ethnographic field notes, the richness of participant observation provided an important first hand perspective. Additionally, having a designated field notetaker with no other role each week ensured nothing was missed. We are confident the participant observation was an asset to our data collection.

We elected not to record, photograph, or film the class for research purposes to protect the privacy of the participants and to avoid introducing a distraction. The class capture system automatically recorded the class for the students’ use, but in keeping with our ethics paperwork, this was not used for research purposes. We felt this was consistent with our confessional ethnographic stance; while video ethnographies exist, they do not film and do not aim to capture all aspects of daily life for later analysis, rather they are a more cinematic technique built on the ethnographers’ lived experience in the field (Redmon 2019). Additionally, extensive recording is ethically fraught given the complexities of power dynamics (Everri et al. 2020), and we felt doing so would introduce a culture of surveillance, undermine trust, limit the very rapport we were aiming to create, and as such, undermine the naturalistic data collection at which ethnography excels.

In addition, robot drivers were asked to complete a weekly 7-question survey, which allowed them to indicate their satisfaction and any technical or social issues experienced when using the robots. The survey data was used to track technical issues and confirm the absence of social conflicts, and no further analysis was conducted for this paper. There was one optional participatory design session halfway through the class, which was attended by 5 people (Jo, Jesse, Nandu, Tasha, and Richard), where students gave interim feedback on their experiences with robots. Finally, all drivers were invited for a 60-minute post-study interview, and classmates who did not drive the robots were invited for a 30-minute interview. 5 Drivers (Olive, Nandu, Xiaoke, Cyrus, Richard) and two classmates agreed to be interviewed. All interviews were completed by the first author. One classmate and one driver asked that they be allowed to give the interview answers in Chinese, a request that our multilingual team was able to accommodate by having a team member translate. The participatory design and interviews were audio recorded and transcribed into English so that the whole team could participate in their analysis. Appendix D presents a detailed description of data collection methods and types for this study.

### Data Analysis

Although telepresence robots have significant benefits for accessibility and disability studies, this is beyond the scope of the present paper. We had many disabled participants, and we will discuss their experiences with their disability in a future publication.

The authors, with the first and second author taking the lead, engaged in abductive qualitative analysis across coding cycles, following Grounded Theory (GT) as a methodology (Strauss 1988; Cole and Gillies 2022) to analyze how identity was mediated by telepresence robots. As of late GT is out of vogue in HCI, and Thematic Analysis on its ascendancy (Brulé 2020). However, methodology cannot be selected in response to research fads. Instead, it must be selected in response to an author’s research objectives. We used the Charmaz/Constructivist (C-GT) approach to GT (Charmaz 2006; Cole and Gillies 2022), which is well suited to theory development from ethnographic data. Here, our goal was not to document qualitative themes regarding telepresence robots in the classroom, such that they could be applied to other domains of telepresence. Rather, our goal was to explain the nature of people’s interaction with robots in the context of a classroom, by putting the evidence from our study into conversation with existing theories in order to extend and develop them. These developed theories could potentially also apply to offices or conferences, but in line with the situated, reflexive character of C-GT, its reinterpretation in other contexts is a matter for future work, rather than unevidenced assertions; the theories developed through GT do not need to prove wider relevance in order to be a contribution in and of themselves. Instead, the theories developed here can be re-situated through further research: while the earliest forms of GT proposed that researchers should avoid engaging with any existing literature prior to undertaking a study as a way to avoid ‘contamination’ by others’ work and thought Glaser and Strauss (1967, p. 37), later work such as C-GT developed more sophisticated ways to recognize the positionality of researchers, acknowledging the importance of engaging with evidence and concepts as a way to improve the quality of observations Charmaz (2006). Indeed, Burawoy (1998) argues that engaging with theory is a key aspect of reflexive research, and both Rode (2011) and Dourish (2007) have criticized ethnographies in HCI for failing to engage with it. Dourish goes as far as to write, “the most useful strategy when engaging with ethnographic work is to ‘read for theory’ as much as for empirical evidence, since these may, in the end, be where the truly significant implications lie” (Dourish 2007). Thus we felt GT was the most appropriate method for our data analysis, and we encourage our readers to “read for theory,” as much as empirical evidence from this study.

An important consequence of this approach relates to the discussion and interpretation of evidence. Unlike positivist GT, Constructivist GT contends that themes and theory do not ‘emerge’ from the data, but are instead co-constructed by the researcher and participants (Cole and Gillies 2022). For this reason, it is impossible to make a neat separation between presenting and discussing findings; ‘presentation’ involves selecting and organizing evidence, which are interpretive and rhetorical moves. Consequently, some arguments pointing to implications for design or practice are made alongside the presentation of the evidence that justifies them rather than reserving these interpretations to a separate section.

Our data analysis consisted of three rounds of analysis of the field-notes, one participatory design session, and interviews. Our use of C-GT included writing code and integrative memos to allow us to arrive at our central theme (Emerson et al. 2011). In the first round, four members of our team open-coded. We removed redundant codes, and what Brulé (2020) calls “bucket codes” - overly general topics created by more junior members of the team doing grounded theory for the first time. We arrived at a combined list of 45 themes, exploring the challenges in using a telepresence robot effectively and appropriately in the classroom (see Appendix A). Prompted by memo-writing (see below), some of our codes were inspired around existing theory such as Dourish and Vertesi’s embodiment, and Murray’s agency, immersion, and transformation. In the second round, these categories were axially coded, generating eleven larger themes (see Appendix B). For instance, open codes such as looking around, acknowledgment, getting attention, and greetings were grouped into a larger category of courtesy practices. Similarly, manipulating movements, video, audio, zooming in, connection issues, battery issues, and sharing materials were grouped into a category of articulation work. Code memos were written that reflexively explored the meaning of each theme. Other ethnographic memos allowed the team to explore how their positionality, the nature of their relationship with the participants, the rapport they developed, and the existing theories available to them to draw on all played a role in interpreting the evidence.

Other team members then commented on memos based on their own fieldwork experiences, and these additions were integrated to strengthen the memos and the coding. Coding memos allowed us to explore the appropriateness of combining some of our initial codes. As these were combined to form axial codes, integrative memos were written by the first two authors, providing reflexively explorations of the meaning of these new themes. These moments, in particular, were points at which the relevance of existing theories was considered. For example, integrative memos were written for the axial codes of “embodiment”, “handless feeling and personal space”, and “courtesy practices”, recognizing both the grounded importance of these in the data we were working with, and the resonance of these codes with existing theory, such as the work by Dourish (2001) and Murray (1998). Finally, we stopped when we reached theoretical saturation, the point when no new insights could be revealed by analysis. Some themes within this analysis, such as best practices for teaching with telepresence robots and issues of disability, were declared out of scope for this paper, and instead will be foregrounded in other publications in education and disability studies (Zhang et al. 2025).

## Findings and Discussions

In the following section, we will discuss examples of how participants engaged in articulation work and courtesy practice in the classroom. We will then discuss immersion, agency, and transformation and finally the successes and breakdowns in achieving them. This section is structured around seven of our 11 codes. We reserved the other axial codes covering disability and teaching for papers targeting the disability studies and education communities.

### Articulation Work in the Classroom

Articulation work for the study manifested in many forms. While articulation work for telepresence has been discussed elsewhere (Elmimouni et al. 2024), it took on a unique significance in the context of ongoing classroom interaction. This aligns with classic CSCW arguments that supporting “the articulation of distributed activities” is a core problem for the field (Schmidt and Bannon 1992) and that minor breakdowns often require substantial, situated coordination work to keep collaboration moving (Grinter 1996; Strauss 1988). In this setting, failing to manage articulation work effectively could negatively impact students’ academic experience. As a result, the teaching team bore responsibility to ensure smooth operation. Although educational risk could have raised ethical concerns, it was mitigated by the fact that students could switch to attending class remotely via Zoom at any time.

Many students reported that, when managed well, participating via telepresence robot provided a higher quality AV experience than attending via Zoom alone. This was due to certain shortcomings in the AV setup of our classroom, which we will address later.

The articulation work that occurred in classrooms was similar to what has been seen in offices and homes, logging in and out, managing the robot’s movements (especially getting stuck on objects); and troubleshooting technical issues like audio-visual problems, connection drops, and battery depletion. However, these took new forms given the classroom context. Thus, next we will discuss specific challenges stemming from keeping the robot charged in the classrooms, handling the logistics of the classroom, managing one’s AV, and managing the materials needed for class. All of these issues needed to be managed both technical and socially to ease over difficulties using one’s robot.

#### Keeping Robots Charged

While the difficulty of keeping robots charged has been discussed elsewhere (Elmimouni et al. 2024; Rode 2018; Neustaedter et al. 2018), doing so in the classroom context led to additional challenges. We certainly had issues that resulted in the Double 3 and Ohmni Pro robots running out of battery during class, necessitating careful checks the night before that the robots were on the charging docks, and bringing chargers downstairs to allow users to top up their charge during class. The robots were charged in our lab space which required moving them 2 floors down to the classroom. Driving the robot to the classroom took a few minutes. We had escorted drivers in case of network issues, (especially in the elevators as we were unable to get Wi-Fi installed there until late in the term), to open the fire doors and press elevator buttons. While some students (Richard and Nandu) valued that arriving a few minutes early, gave them a chance to interact with their classmates and professor, other students (Cyrus and Tasha) felt the process was time-consuming and difficult to manage in the context of a full-time job. Additionally, both Cyrus and Xiaoke’s expressed concern that they could not safely drive the robot. Cyrus and Xiaoke both asked us to carry the robot downstairs for them, which was possible given the selection of a lighter form factor robot. While having us move the robot might have been beneficial from the student’s perspective, by not moving the robot themselves, the students had less opportunity to practice driving somewhere safe and less opportunity to socially integrate themselves in the classroom. Thus, whenever possible, we recommend encouraging students to enter the classroom under their own power, promoting agency and social connection. Additionally, doing so is logistically easier, physically moving the robots to the classroom is much harder than escorting them them through a building. In the long term, we hope to see students using the robots for multiple classes, and for engaging in social experiences between them. However, moving docking stations directly into classrooms is not a practical, long-term solution for a more integrated campus roll-out.

#### Classroom Logistics

Additionally, some forms of articulation work were unique to this classroom setting, especially networking in an international educational context. Networking issues were complicated by some students connecting from elsewhere in the the UK (Olive, Jesse, Xiaoke, Tasha), while others connected from the Global South including India, Thailand, and the United Arab Emirates (Nandu, Richard, Cyrus). Our students encountered issues with maintaining a stable connection despite high bandwidth connections and use of VPN. Such problems highlight how hybrid participation depends on infrastructural conditions that are uneven across locations and cannot be assumed as stable “background” resources (Star and Strauss 1999).

**Fig. 2:**
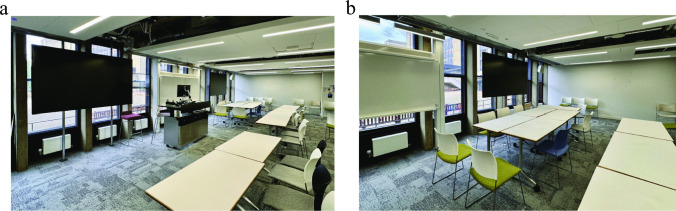
(**a**). Classroom Layout and (**b**). Close-up photograph of the area of the classroom used for this study. Note a table for 10-12 people was used to facilitate small group discussion

A great deal of the articulation work stemmed from the imperfections in our classroom. Due to room booking logistics, we were unable to book a classroom designed for a hybrid class. Thus, we were in a classroom that had the cameras focused primarily on the podium. There was a room video, but it was intended to capture the entire long, narrow 100+ capacity classroom, rather than the small table where less than 10 people sat (see Fig. 2). Further, speakers in the room projected the voices from Zoom. However, there were no tabletop microphones, and the provided portable microphones were clunky as passing them around slowed down discussion, and people struggled to hold them at the proper distance from their mouths.

Lastly, there was a long umbilical cord with video, audio, and power from the projector to the professor’s laptop. This, along with chairs, coats, bags, laptop cables, and robot docking station power cords created numerous obstacles in the classroom. To foster a conducive teaching environment with extensive educational technologies, collaboration with learning environment designers is essential to improve the construction and operation of learning facilities.

#### Managing One’s Video and Audio Quality

Managing audiovisual quality formed one of the most complex forms of articulation work. All but one of our telepresence users also elected to log into our learning management system alongside using the telepresence robot, though notably Nandu in his second week elected only to use the robot and not Zoom. This is akin to Neustaedter et al. (2016)’s finding that a back-channel for communication was needed amongst telepresence attendees of conferences. For instance, “Xiaoke contacted me by WeChat. She said she was sad that she can’t hear the class clearly. (I assumed although she used “sad” in her text, it’s not a super upsetting emotion, that in Chinese context we sometimes use it in daily conversation to express mild feeling... a bit frustrated)” (FN-RA3-W8). Our learning management system gave students an additional shared text chat, but also afforded them the chance to present via video and audio. Thus, telepresence users, by manipulating their microphone source settings, could manually choose to project their audio through either the room speakers or the robot speakers. Similarly, this meant that they could listen to the audio stream from the Zoom audio, from the robot directly, or both. From the standpoint of the people in the class, this meant that sometimes they would hear a student speaking from their robot’s speaker, and at other times, despite the student’s visible presence on the telepresence robot’s screen, they might hear their voice from the corner of the room. By all accounts, this felt awkward. Novice robot users appeared to rely on a single audio or official source and often expressed frustration that they could not hear (Tasha, Olive, Cyrus). Richard adopted a complicated practice to compensate: when the speaker was near the robot, he used the robot’s microphone, and when the speaker was far from the robot, he used the room microphone. To accomplish this, he listened to one audio on headphones and another via his computer speakers. This resulted in an awkward dance involving him taking on and off the earphones, which prompted curiosity in our field notes:

Richard asked if he could ask a question. He changed the volume as he asked to make it louder. This time, he was very clear, and slightly louder than the students in the room... [Prof] and Richard continue the conversation; Richard has his headphones off at this point and appears to be leaning in towards the camera through this discussion, taking up more of the screen. When the conversation moves on, Richard puts his headphones back on. (I wonder why it was better for him to wear these most of the time, but not during this exchange with [Prof]). (FN-VProf-W7)

Cyrus similarly mentioned the difficulty of managing two audio streams,“I felt there was some improvements made with about the sound and audio in general, but that meant me having both Zoom going on at the same time and the robot going at the same time. It was kind of like a compromise between uh, you know, the two solutions.”(Cyrus-Debrief). By mastering this complex articulation work, students were more successful in being able to hear what was going on and consequently meaningfully interact with the rest of the classroom. The more sophisticated users could take advantage of a mixture of telepresence and conventional classroom technologies to maximize the quality of their audio and video, but this required considerable technical skill. Future classroom management systems should be designed to allow telepresence users to easily toggle between video and audio sources integrating the UI of the robot into the classroom management system.

#### Managing Materials: Windows and Papers

Students experienced window management issues, as students had to manage the user interface for the robot, Zoom, their source papers for the discussion, which were largely electronic, and their class notes. Students did this in the context of their home technology setups, which meant that some required additional monitors (Richard), or tablets, and some, like Olive, tried to do everything from a single small laptop screen. Additionally, there were issues in sharing digital and physical materials (books, journal articles, and field notes) between corporeal, Zoom and robot-based students; this often required the use of Zoom features such as screen sharing, and the document sharing feature available on some robots. Participants frequently relied on features such as screen sharing, visualizers, and document-sharing tools, consistent with prior research showing that hybrid participation depends on making artifacts visible and referable across heterogeneous media (Jakonen and Jauni 2021). For instance, one week when Olive was attending in person, she was discussing her field notes, showing the physical document to the class. The Professor realized the students using robot and Zoom could not see them. “[Prof] then held up the laptop so that Cyrus could see what was being shared via the Double Camera. There was then a tricky moment for hybrid participation when showing this to the people on Zoom using the [classroom’s] visualizer camera, where the display lost the camera feed for the participants.”(FN-VProf-W3). Future classroom management systems including telepresence robots must easily facilitate window management of both notes and the articles being discussed.

#### Conclusion: Articulation Work

Historically, articulation work has had a political dimension, drawing attention to the invisible labor needed for individuals to fit within structures of projects, institutions and so on. Our observations were filtered through the established power dynamics of the classroom; we did not see articulation work unfold in terms of broad political dimensions, but rather through the more banal aspects of articulation work, such as the patterns of extra work required by the teaching team to maintain the technologies students used. While many of our findings regarding batteries and WiFi were not radically different from findings that could arise in offices or at academic conferences, to make telepresence robots usable in the classroom requires a deep understanding of how these issues are made manifest in academic contexts, thus their focused study is vital.

Ultimately, the situation in the classroom was fluid, accommodating many part-time students whose professional commitments would necessitate various participation modalities. Additionally, in a post-COVID world, we had students who caught the virus and wanted to isolate. As such, who was participating in what fashion was fluid. Students need to plan ahead if they need to install software on computers managed by their employers. To facilitate easier classroom management, we recommend mandatory training at the start of term for all students, even those who only plan on using the robots due to illness. This training should cover all of the different robot modalities and be customized to specific types of articulation work we witnessed. Clear and effective training can reduce the amount of articulation work required. However, when articulation efforts failed, our students would cover their faux pas by enacting courtesy practices. One could even go as far as to argue students engaged in a new type of articulation work, courtesy work, to preserve the norms and power dynamics of the classroom.

### Courtesy Work in the Classroom: Traditional and Emergent Courtesy Practices

In our class, many traditional courtesy norms, as conceptualized by Brown and Levinson (1987), continue to play an integral part in facilitating social interaction and cohesion. However, these norms often require recontextualization and adaptation to fit a technologically-mediated setting. We will argue that these adaptations to help smooth over articulation work have gone awry. This section will critically examine the preservation and transformation of courtesy norms as we know them, focusing on instances of greetings and introductions, apologies, and strategies for active listening and participation.

#### Greetings and Introductions

Greetings and introductions are foundational elements to build social rapport, setting the stage for future interactions. In settings with telepresence robots, these norms are often adapted to wallpaper over gaffes created by telepresence technology.

Firstly, telepresence robots redefined the norms for greeting in the classroom by emphasizing the need to reaffirm presence when rejoining the classroom. Unlike in face-to-face settings, where brief absences such as stepping out of the room often go unremarked, telepresence made such absences more pronounced due to the disruption caused by technical breakdown. For example, when Richard, attending via the Double 3 robot, was disconnected due to a technical issue, the class instructor attentively awaited his return and promptly welcomed him back with a warm, “Welcome back, Richard.” (FN-Olive-W8). This example illustrates how telepresence technology redefines the concept of “being present” in the classroom. The instructor’s deliberate acknowledgment of Richard’s return was more than just a courteous gesture; it was essential to reestablish his presence which was temporarily interrupted by the technology.

Our data highlights two aspects of greeting that go well beyond what is discussed in the prior literature. We report these adverse events in the spirit of Rukmane et al. (2022) emphasizing the need to discuss failures, and by extension adverse events, as a learning opportunity. We saw examples of discourtesy, people not in the class pointing at the robots or calling them “weird” (FN-RA2-W9), or letting a door slam in the robot’s face rather than holding it (FN-RA2-W10). In another instance, this discourtesy became harassment. At one point, a Chinese student was driving the robot and was greeted by a construction worker: “I observed a white man who looked to be in his 30’s/40’s working in the area randomly saying ‘ni hao’ [to student] despite not knowing specifically her ethnicity was, what language was her mother tongue, or even what languages she spoke.” (FN-RA2-W2). Thankfully, the student did not hear this utterance, which was reported to university management as an instance of racism since it constitutes a known microaggression. This is because this interaction reduces the student to a racial stereotype based on assumed appearance and presumed foreignness. This kind of interaction can be alienating, reinforcing power dynamics that mark out the student as different or out of place. This incident is akin to Elmimouni et al. (2024) who talk about the problems of students being dehumanized while using robots, and this incident highlights the need for teachers to be vigilant against bullying.

Another more heartening example of a courteous greeting occurred when VProf’s cat came on screen. This prompted concern that the cat could drive the robot which has been reported elsewhere in the literature (Rode 2018), thus he writes “I wondered if there was a way to lock the movement function of the robot, in case he [the cat] accidentally activated anything.” Shortly thereafter during a break, the TA, the professor, and several students went over asking to meet the cat, “So I moved the laptop so he was on camera, and unmuted it. He [the cat] started mewling at everyone. [The TA] suggested I let him drive the robot, but I explained that I’d disabled the keyboard to make sure he couldn’t” (FN-VProf-W3). In this instance, the class, by acknowledging and welcoming the cat and making jokes, set VProf at ease despite his anxiety about the cat accidentally controlling the robot. In this way when the articulation work proves inadequate, they are made up for by courtesy practices around greeting.

#### Apologies

Apologies, as acts of courtesy, help to “repair breaches in social etiquette” (Kort, 1975, p. 80). The use of apologies also took on a special significance in our classroom, reflecting many communication challenges through telepresence technology. Apologies addressed unique challenges in telepresence settings, particularly by acknowledging and mitigating the social discomfort and power dynamics such as dominance caused by the limitations of technological embodiment. For example, Richard’s apology for “towering over” his classmates while attending via a Beam+ robot: “I feel as if I’m towering over you, so I’m sorry” (FN-Olive-W6). This highlights the limitations of telepresence in replicating the nuanced human interaction such as the ability to adjust one’s posture or positioning in response to social cues and the potential social discomfort it can induce. This apology addressed the social discomfort caused by imperfect embodiment and perceived dominance.

Additionally, the need to apologize for technical issues, events often beyond the user’s control, emerged as a unique form of courtesy within this particular setting. For example, when Cyrus, driving the Ohmni Pro, accidentally disrupted a conversation between the professor and Nandu with an unintelligible noise, he instinctively apologized (FN-Olive-W8). This exchange implied that while traditional courtesy norms were maintained, they were also being reshaped to accommodate the existence of telepresence, where users were accountable for disruptions that stemmed, in part, from the technology itself.

In digital spaces, the need for such apologies adds another layer of accountability. Unlike in-person interactions, where many disruptions can be attributed to human error or misunderstanding, disruptions caused by telepresence robots are often outside of the user’s direct control. Yet, the social expectation remains that users take responsibility for these disruptions, as evidenced by Cyrus’s apology. Overall, this microcosmic interaction reflects a broader societal trend in which individuals are increasingly held accountable for the actions of the technologies they use, raising questions about the fairness and implications of such expectations. These apologies for technological mishaps also reveal an underlying tension between human agency and technological determinism. While users like Cyrus and Richard apologize as if they are fully in control of their actions, the reality is that the technology mediating their presence introduces elements of unpredictability, demonstrating the complexities of maintaining traditional courtesy norms in environments where boundaries become blurred.

#### Performing Engagement: Active Listening and Participation

Active listening, which involves demonstrating elements of engagement with a speaker, was another critical aspect of positive politeness (Brown and Levinson 1987) demonstrated in our class. Throughout the course, Cyrus’s frequent nodding through his screen (FN-Olive-W3) and Richard’s verbal affirmations, such as “mmm” during discussions (FN-Olive-W5), closely mirrored in-person behaviors, demonstrating that even mediated by telepresence, participants could maintain conventional active listening practices. However, the limitations and affordances of telepresence robots also necessitated the development of new courtesy practices in this context.

New courtesy practices involve various forms of articulation work that demonstrate attentiveness and responsiveness, which we dub “courtesy work.” One emerging practice involved the deliberate manipulation of the robot’s camera to simulate non-verbal cues, like open body language. Often, students chose to turn their telepresence robots to face speakers directly, even though some of the robots had wide-angle cameras that could capture large portions of the room without requiring drivers to move and scan physically. For example, Cyrus intentionally turned his robot to face Tasha while she spoke via Zoom, and after that, when the instructor started talking, he turned his robot to face the instructor and the teaching table (FN-RA2-W3). We also see examples in Week 2 when, “Nandu, who was already facing Dan, moved with a simple shake when Dani mentioned his name during her introduction, indicating responsiveness” (FN-RA2-W2). These examples demonstrate how students actively manage their telepresence robots to enhance interaction and maintain a sense of engagement to show politeness to speakers in the classroom.

Additionally, another courtesy practice is to make responsive adjustments to the screen on telepresence robots. For example, drivers frequently adjusted the robot’s screen height to approximate eye contact. When Richard lowered the robot’s screen to meet the gaze of seated participants, Cyrus perceived it as an intentional effort on his part to maintain eye-level communication (FN-RA2-W3). We did not see Cyrus adjust the Ohmni’s pivot as he had less time to develop fluid control over that robot than he had with the Double. However, engagement and courtesy practices extend beyond just the humans who were using robots. Humans present in person, including students and the instructor, also play vital roles in fostering comfort and politeness in a co-presence context. For example, in an effort to make Olive feel more comfortable, RA2 chose to kneel while speaking with her rather than standing, which could have been perceived as imposing (FN-RA2-W4). When his knees began to hurt, RA2 communicated with Olive about his need to get a chair and sit down. These deliberate actions demonstrate that participants are aware of the importance of courtesy practices, such as open body language and eye contact, in expressing attentiveness, even in a technologically-mediated setting.

Moreover, the aforementioned emergent practices reflect a deeper understanding of the communicative affordances of telepresence robots, despite their limitations in offering the full spectrum of human non-verbal communication. The ability to adjust the robot’s body orientation and screen height allowed drivers to consciously choose how to present themselves in relation to others–actions that might be less deliberate in face-to-face settings.

#### Conclusion: Courtesy Work

In conclusion, the maintenance of traditional courtesy norms in our class, as facilitated by telepresence robots, reflects the complicated relationship between continuity and adaptation. While elements of greetings, apologies, active listening, and participation in this context were preserved, others required recontextualization that acknowledged telepresence’s unique advantages and disadvantages. The adaptation of these practices points to the evolving nature of courtesy, where traditional norms are not just replicated but are actively redefined and reconstructed to meet the demands of a new educational and communication landscape in response to articulation work. Ultimately, this section presents the argument that while telepresence technology can support the maintenance of social norms, it also introduces new dynamics that require a rethinking of how these practices are enacted in technologically-mediated environments. We call these practices “courtesy work”, and argue they represent a form of articulation work in response to the pressures of a telepresence robot, allowing users to navigate the political realities of a classroom, including its norms and power dynamics.

Courtesy, as we will show, next ties to embodied interaction in that when technology becomes an obstacle, courtesy becomes harder to enact. However, when technology becomes invisible, courtesy flows more naturally, mirroring in-person interactions. We will next turn to discussing embodied interaction and transformation.

### Achieving Transformation in the Classroom

As we have shown within telepresence-mediated environments of our classroom, articulation and courtesy work proved barriers to embodied interaction. To better understand these dynamics, we will discuss three key goals for embodied interaction drawn from Murray’s theory that proved productive in the analysis of our data: achieving immersion, having agency, and achieving transformation. We will explore each of these goals in turn.

#### Immersion

Immersion, as defined by Murray (1998), requires the user to feel fully present in a remote environment. Like Kornfield et al. (2021), an important part of this was feeling “really there,” but Kornfield’s discussion was not grounded in the theory of Dourish and Heidegger, and we wanted to explore what Dourish’s use of Heidegger’s concepts added theoretically to our understanding of successful mediation of educational experiences. We see an instance of immersion with Richard when using a telepresence robot located between the small table and the projection of the learning management system.

[Richard]...initially had his back turned to the screen to participate in the round-table discussion. However, when the meme [image] was shown [by the professor], he turned his robot 180 degrees to view it directly. Moments later, he said, ’Sorry, I’m blocking your view, everyone,’ and... quickly moved his robot from its position in front of the screen and settled it into the nearest corner of the room, still facing the big screen. (FN-Olive-W6)

Richard’s quick apology and repositioning of the robot exemplify how embodied interaction can facilitate courteous behavior. In this instance, the technology was ready-to-hand, allowing him to focus on the dynamics of social interaction rather than the mechanics of controlling the robot. The flawless execution of this courteous act highlights the need for immersion when engaging in polite and socially appropriate behavior in a digital-physical hybrid environment.

Immersion was broken repeatedly in our study through bad connections (Richard), dead batteries (Cyrus, and Richard), poor audio (Cyrus, Olive, and Richard), and generally frustrating experiences (Olive, Tasha), due to the cumulative effects of articulation work. For instance, in the last week, during the students’ final presentation, a well-intentioned attempt at immersion –inviting Cyrus and Richard who were both attending via telepresence robots to go up to the podium in the classroom– proved problematic. Access to the podium was blocked by a wheeled stool intended for the professor, who, ironically, was also attending via a robot. Robotic professors do not need stools, they need stools not to block the podium. Our TA quickly moved the stool away, but the immersion had already been broken. At that moment, Richard and Cyrus were cognizant of their handlessness (Rode 2018), notably their inability to manipulate physical objects, breaking the sense of immersion. Immersion is fragile, yet it can be sustained through careful attention to articulation work and courtesy practices. Ideally, the course staff could have anticipated the problem and removed the offending stool out of the way sooner, or the students could have asked. But anticipation is hard. What is more, as Richard was driving a robot without collision detection, he could have pushed the stool himself, thereby demonstrating agency.

Still, we see repeated examples of immersion, such as Nandu and RA2 playing tag, or during a break, when Richard and Nandu took advantage of their robots and both independently stared out the window from their respective robots, feeling a connection with campus life as they reported in their debrief interviews. Similarly, Richard demonstrated immersion when he was halfway down the corridor driving to class, and he said, “It’s this way right?”, and later recognized and approached his classmates across the lobby of the building en route to class (FN-RA1-W6). Here Richard, despite being located in the UAE, had achieved sufficient immersion to develop a mental map of the multi-floor journey from our lab to the classroom and to recognize classmates outside of the usual context.

Finally, immersion is not an all-or-nothing experience, as illustrated by the harrowing incident of Richard and RA2 getting repeatedly stuck in an elevator taking the telepresence robot, piloted by Richard, back to the lab. Please note that no students were hurt in this incident, and the problematic elevator was immediately reported to the campus facilities. Richard was safe in the UAE, so he could have just logged out, but he stayed throughout the incident. RA2 wrote, “After about 2 minutes [of being in the elevator] I realised the doors had not opened, and started to panic, I pressed buttons and banged on the door, eventually the door opened”W7), thus Richard likely hung around as his classmate was struggling. Finally, the doors opened, and RA2 noted, “I instinctively walked out, but fell slightly, [as] it turned out the elevator was higher than the floor’s level by a few feet, due to a malfunction and I didn’t notice. I quickly tell Richard to wait, and not to come out. Richard seems shocked but listens.” They later realized the door opened on the wrong floor and tried the elevator again, largely because the robot was too heavy to carry up the stairs and, they were likely too upset by incident to consider that re-entering the same elevator was a bad idea. They promptly got stuck again, and the elevator started to shake, “The juddering continues, and I am freaking out. I say to Richard, “You might watch me fall to my death via the telepresence; I guess that’s good data,” and he assures me that these lifts are very secure and won’t break, but it sure... didn’t feel like it.” Richard was both immersed and not immersed in this environment. His physical body was not at risk, and yet, had RA2 not cautioned him, he might have driven his robot body off the edge to fall “a few feet,” jeopardizing his robotic embodiment for class. The RA was clearly frightened, and Richard was immersed in that panic enough to comfort his classmate. Further, by the RA raising the specter of the elevator falling and killing him, Richard was cognizant that should a disaster occur, he would have to watch without the power to intervene. At that moment, the immersion was broken due to his lack of agency.

#### Agency

Extending Murray’s Agency in this context, agency is achieving control over one’s remote environment. While the elevator example shows a lack of agency, in other instances, we see students exhibit it. For instance, in one class session, Xiaoke demonstrated an embodied interaction through her telepresence robot:

[Prof] then asked [Olive] what I found notable from the week’s reading... In preparation for my response, Xiaoke turned her robot approximately 30 degrees in my direction, so that she could face me directly while I spoke on authenticity, plausibility, and criticality. Once I had completed my response, [Prof] next called on Xiaoke, asking her to read a passage. Without hesitation, Xiaoke angled the robot toward [Prof] before responding. (FN-Olive-W8)

Here, the robot was ready-to-hand, allowing Xiaoke to maintain open body language and participate fluidly in the discussion. This readiness resulted in smooth, courteous interactions that closely resembled face-to-face communication.

Conversely, we see examples of lack of agency. At one point, during the AV setup for class, Prof was discussing with the students and research team how covering one’s camera is a personal violation. To illustrate her point, she, having a good rapport with her friend VProf, quickly passed her hand in front of the camera. This instance prompted the following field note, “it was a bit strange, like seeing a hand emerge from my stomach in a funny way rather than body horror. We both laughed.” (FN-VProf-W3). Later, when the research team were moving robots downstairs to the classroom, quarters were very cramped with several people plus robots trying to leave a small office for class. The Prof went to throw out a can across the office into the trashcan, playfully tossing it through the two stems of VProf’s robot supporting its head. VProf reported, “it was a bit like having someone throw something through my legs, but in a very non-threatening way, like a friendly trick-shot in a game” (FN-VProf-W3). This aligns with Kaerlein (2012) who emphasizes that robotic bodies, by facilitating immediate and communicative channels, enhance the sense of “presence.” In this case, Beam Pro’s two legs mimic those of VProf, bridging the gap between virtual and physical presence. Later in class, when discussing best practices for writing field notes, Prof wrote,

This got really meta. It hurts my head writing field notes about teaching about field notes...I started talking about how you wrote [field note] asides in () with analysis and reflection and that these emerged into analytical memos and those and field notes ended up being included in formal writing. I needed an example. Robots!... . At some point I told them the story of [VProf] leaving my office. I pointed at the where I had thrown the can, but I didn’t stick my hand there. Its seemed like far too much of a violation. Partly for comic effect (got to keep the students amused) and partly for making the point about how field notes needed to talk about appropriateness around robot-mediated touch. I pointed out how it was possible to block a camera and how that wasn’t okay. (I got worried that [VProf] found it too disconcerting). I think I heard a gasp. It was very clear that students were having visceral reactions that this was not okay. I felt terrible, but the rules of courtesy behavior are unknown here. (FN-Prof-W3)

This likely stemmed in part from the students being unaware that the professors were friends and playfully troubling the norms of propriety in trying to discover them. Part of this likely also stemmed from the viewpoint, as Prof was behind VProf, whereas Jo was in front of him. Jo took the opportunity to explain what she thought was going on:

Jo pointed out that throwing a can through the middle of the robot seemed like throwing something between [VProf]’s legs and that wasn’t an okay thing to do. I got embarrassed and realized that we were basically mapping the robot body to a human body, I would have never done that in real life. (FN-Prof-W3)

In private conversations, Jo mentioned she initially perceived it almost as a grope, which resulted in frantic apologies and explanations between colleagues. This instance illustrates how practices of courtesy and agency can emerge unexpectedly.

Takayama (2015) discusses the problem of attribution of agency to the user or the robot, resolving this required articulation work. Thus, in response, we found an emergent courtesy practice around the appropriate touch of the robot to preserve agency. For instance, when RA2 was concerned about Cyrus’s battery life, he touched Cyrus’ robot to see the battery life indicator. He commented, “I quickly realized this a violation.. of the social norm not to touch a robot without the user’s permission” (FN-RA2-W4). While his intention to provide technical support was well-meaning, he inadvertently compromised his charge’s agency. All students, except Olive given her dual role, avoided touching the robots entirely. Through discussions of Olive’s field notes in class, a few norms emerged:

- If possible, do not touch a robot without permission.
- If not possible to seek consent, only touch the robot to help its user. e.g. to charge the robot, place in a space with Wi-Fi, or untangle it.
- If touching the robot try and touch the bezel of the head. Touching the stem was a violation of personal space.
- Avoid covering cameras as it was akin to blinding the robot user, avoid blocking or leaving obstacles in the robot’s way as that immobilizes it.

These norms were confirmed in debrief interviews with participants and were consistently agreed upon. This was akin to the social norm recommendation in Elmimouni et al. (2024), where they called to refer to students using robots by the student’s name, rather than address them as “the robot.” Additional work is required to explore best practices for ensuring the comfort of robot users in the classroom.

While controlling one’s embodiment helped, the sheer amount of required courtesy and articulation work was often overwhelming. Cyrus became so frustrated that he stopped using the robots in Week 5 and only gave telepresence another try when we acquired a new Ohmni Pro. Olive in her field notes passionately discussed her lack of agency in trying to control the “unruly, unwieldy, and unpredictable” telepresence technology using a metaphor of sci-fi “body snatching,” complaining of the inherent tension with her desire to adhere to the social norms of the classroom, writing,

Despite my efforts to control its body, I was constantly reminded of the fact that it was not mine, rather a surrogate with my face. This body often resisted my commands, leaving me feeling, pun intended, alienated—not only from myself but also from my classmates. (FN-Olive-W4)

Note that Olive’s reference to her robot body as “it,” coupled with her use of the word “alienation,” underscores her lack of ownership. She struggled with the articulation work,

While my mouth was moving, the class looked at me with furrowed brows... They couldn’t hear me. On the interface, I muted and unmuted myself repeatedly. Regardless, my classmates still couldn’t hear anything... I could feel my frustration growing and became worried that [the profs’] would grow, as well. (FN-Olive-W4)

She shares how she was so frustrated with the robot that she uncharacteristically refused to answer the professor’s question when called on, despite Olive’s American Southern pride in being polite:

However, in saying no, I not only invalidated the work I had done to prepare [for class], but I also appeared to be actively indifferent to participating in the class. This was not the perception I wanted of myself as a student, but in the moment, I couldn’t help myself. (FN-Olive-W4)

Olive attempted to engage meaningfully in the conversation, but the robot’s technical limitations rendered her voiceless, and without agency. This culminated in a final indignity,

I noticed that my screen started zooming in on [Prof], even though I was sure that I wasn’t playing with my mousepad. Even worse, it didn’t zoom in on her face. No, it zoomed in on her chest... I felt both uncomfortable and embarrassed that I had somehow just violated the social norms of a student-teacher interaction without even realizing it. (FN-Olive-W4)

In this scenario, as in others, the technology was decidedly “present-at-hand,” drawing focus to itself and preventing Olive from maintaining the expected norms of a student-teacher interaction despite her efforts to maintain appropriate boundaries. Olive wrote, “The inability to correct these issues quickly led to a heightened sense of disconnect, where I felt my efforts to be courteous were thwarted by the very tool meant to facilitate their presence in the classroom. The constant struggle to manage the robot created a barrier to maintaining politeness, leading to frustration, embarrassment, and even social faux pas.” Not only did she feel a lack of agency, but she experienced it in a way that violated her sense of self and her culture’s ideals of politeness. Nevertheless, for some of our users, especially Richard, and to a lesser extent, Cyrus and Nandu, they were able to manage the articulation work and engage in courtesy practices to a degree that allowed them some agency.

#### Transformation

For Murray, the highest form of interaction is transformation, and indeed on some rare instances where both agency and immersion were achieved, we also observed the transformation. In the vignette at the start of this paper, for instance, Nandu and RA2 engaged in robot-mediated play, and in another instance, Richard challenged Cyrus to a robot race (FN-RA2-W8). Similarly, in the instance with the visiting professor’s cat, the robot enabled natural interaction between students and the professor’s cat, bridging the physical classroom space and remote space. That said, most of the students did not report experiencing transformation as having occurred. When asking Richard whether he felt the robot was an extension of himself, his response was fairly typical: “I don’t know if it was just the audio feedback, I definitely could have a conversation with Nandu or with [RA2], but it didn’t feel as fluid and as natural as like a Team call or an... person interaction or a phone call. It’s quite clunky if I’m honest with you, robot to robot” (Richard-Posttest). This sentiment was that the technology had not reached a state where interaction was seamless and transformation occurred.

“I feel like my note talking has become normal, and that most of the time, I’ve lost track of the remoteness of my participation- it’s now just specific moments that draw my attention to where I am and am not; things like the viewpoint that seemed odd before have just become taken-for-granted”(FN-VProf-W3). The most compelling example amongst our students occurred when saying goodbye on the last day of class,“[Prof] announced the end of the class. Cyrus, [with] a dramatic final wave, spins around on the spot in class to try and address everyone... Richard does a similar spin” (FN-RA1-W10). In this emergent goodbye ritual, it appears Richard and Cyrus are fully cognizant that this is the last time they will be collocated with their classmates. Our findings showed the relevance of transformation to telepresence.

## Towards a Theory of Transformation as a Goal of Telepresence

Our analysis, working in the tradition of C-GT, involved putting new evidence into conversation with existing theories, including theories developed in other contexts that could be extended or developed to explain experiences of telepresence in education. Specifically, our findings demonstrated the relevance of existing theories of transformation and embodiment to understanding telepresence mediated interaction.

The transformational experiences we witnessed, while fleeting were made possible through our participant observation. They show that if you have immersion, and agency, and your articulation and courtesy work are not too taxing, it is possible to feel like you are really there. This develops the concept discussed previously in Rode (2018) as “handless feeling,” which describes the potential for a magical transformation where you simultaneously identify with both your corporeal and robot-mediated body. Drawing on Dourish’s theoretical uses of Heidegger’s concepts of mediation, in these moments, telepresence technology becomes “ready-to-hand,” and can function as an extension of the user’s body, providing for more intuitive and courteous behaviors. Experientially, when this is achieved, the robot fades into the background, allowing the driver to focus less on articulation work, and more on social interaction. While our data suggest that our participants’ experience with transformation was limited, it also shows that it is possible. Thus, we look to transformation as a design goal of telepresence.

Our C-GT analysis identified technical and social elements that need to be present to achieve this design goal. Articulation work was a central theme, involving infrastructure (batteries, WiFi), video and sound quality, room layout and the coordination of educational materials (e.g. books). Courtesy work is always part of education, but was intensified here by technical issues that breached social etiquette and required apologies, and by emergent situations where norms had to be negotiated as part of the process. Both of these interfered with any sense of immersion, and while these incidents invited agency, this was in the form of social and technical repair work, not meaningful educational participation. The prevalence of these incidents helps explain why transformation was so difficult to achieve.

However, when transformation occurs, telepresence afforded deeper embodied participation than is possible with traditional videoconferencing applications. It made new forms of agency available to remote learners, through the ability to use their mediated embodiment to position the telepresence robot, and to have greater control over how they shared their image, sound and digital resources with classmates.

## Conclusion and Future Work

In this paper, we have presented an ethnographic study of deploying five different telepresence robots “in the wild” for a graduate class. We focus on the student’s experience using the robots and catalog the challenges they encounter in using the robots to mediate their studies. We catalog the types of articulation work and courtesy work. We then discuss how they provide both support and obstacles to the type of transformational experience students want in a remote classroom.

Our study shows that usage practices vary and that future work must explore these differences, especially with regard to user types, robot types, pedagogy, and additional developments for classroom robots. First, the needs of the infrequent robot user who attends class occasionally when ill, are different than the student who uses it long distance for the bulk of their academic career due to being a remote student. Further work is required to look at the differences in these user needs. In particular, the needs of disabled students will need to be considered, as students with disabilities that make travel difficult will be especially drawn to telepresence. Secondly, additional research is needed to examine the differences between telepresence robots and which manufacturer’s designs are best suited to an educational environment. Third, future research needs to investigate pedagogical practices. Best practices for robot user training must be developed carefully, including how to best include students using robots in classroom activities and what types of activities are feasible and appropriate. Finally, the robots themselves are presently ill-suited to classrooms, and much could be done to improve their interfaces. Robots must be able to safely drive over the detritus of book bags, coats, and laptop cords on a classroom floor. The robots must be integrated into teaching management systems, allowing students and professors to manage the various audio and video streams, as well as participate in discussion back channels. More support is needed for reviewing readings, lecture slides, and material provided spontaneously by classmates.

However, despite the need for much future work, in this paper, we used constructivist grounded theory as a methodology to extend existing theory around embodiment and transformation in order to explore telepresence mediated communication practices in education. We argue that robot-mediated identity work occurs with the goal of transformation–a goal that requires, but goes beyond, Dourish’s concept of embodied interaction. We discussed a host of articulation work–including driving the robot to the classroom or having the teaching staff bring it down, managing the process of logging in and out, managing movements, getting stuck on objects, audio, and visual issues, network connection, battery issues, and using the robot in conjunction with our learning management system. We argued that courtesy practices emerged in part to wall-paper over the inability to adhere to the social norms of propriety in the classroom when robots made unexpected noises, logged out intermittently, and made it difficult to participate in class discussions. We discussed how courtesy norms for greeting, apologizing, active listening, and participation were practiced by the students. We also discussed emergent norms around computer-mediated touch. These articulation and courtesy work practices often occurred to repair breaches in social conventions, for example, due to technical issues; however, when used agentively instead as an expressive form of participation, they allow students to achieve transformation and to feel like they were really in the classroom despite their robot-mediated body.

Engaging with Murray’s transformation theory, we argued that both immersion and agency were both necessary (but not in themselves sufficient) precursors for transformation. Thus, we cataloged from our field notes examples where students experienced or failed to experience immersion and agency. More research is needed into how to sustain immersion and agency in telepresence, but these field notes are the starting point for establishing strategies for students, pedagogical shifts for teachers, and changes to telepresence technology to better facilitate immersion and agency in the classroom. Ultimately, the question is whether robot-mediated interaction is appropriate for hybrid classes. Our students had truly mixed experiences with telepresence, but our data suggests that in some instances, transformation was achieved, and the robot was truly experienced as an extension of the user’s self, a tool that was “ready-to-hand.” While a host of improvements are needed to ensure better telepresence experiences, our study’s core contribution consists of showing the promise of robotic telepresence, the potential of feeling like you are really there in a classroom despite being half a world away. After all, the promise of transformation was so tantalizing that our students elected to use them week after week, demonstrating that they saw enough value in telepresence, despite all the articulation and courtesy work to keep choosing to use them despite having conventional remote meetings tools at the ready.

As discussed in the literature review, conventional remote meeting technology leaves users feeling a lack of presence and is generally preferred less than face-to-face education. Given that some students with disabilities and international students from lower socio-economic backgrounds will continue to find the costs and logistics of in-person education problematic telepresence will continue to be the best next alternative. Future work must help minoritized students realize this dream of transformative telepresence education.

## Data Availability

No datasets were generated or analysed during the current study.

## Appendix A Summary of Open Codes

The following 45 open codes were generated by the four coders:

- looking around
- handless feeling
- immersion
- agency
- transformation
- present at hand
- greetings and introductions
- violation of social norms (failure to greet, not looking when talking)
- acknowledgments
- getting attention
- adjusting volume level
- muting
- adjusting speed
- adjusting height
- touch
- social connection
- robot as spectacle
- difficulty identifying driver
- asking questions
- asking for permission
- privacy
- choosing where to sit
- not moving- silent and still
- apology
- active listening
- navigating and managing space
- toggling between video streams
- toggling between audio streams
- getting stuck
- zooming in using robot
- network connection issues
- battery charge issues
- looking out window
- robot stability
- starting class
- logging off
- logging on
- sharing materials
- peers helping drivers
- getting formal technical support
- teacher practices to include robots
- training
- mental health
- neurodiversity
- physical disability

## Appendix B Summary of Axial Codes

The following 11 axial codes were produced:

- traditional courtesy practices re-enacted with robot
- emergent courtesy practices using robot
- articulation work
- embodiment
- immersion
- agency
- transformation
- disability related articulation work
- telemediated disability presentation
- teaching practices
- additional training required

## Appendix C Additional Information on Data Collection: Number of Students (On-site and Off-site) and Participant-Observers by Week

**Table 3:** This table lists the number of students attending on-site and off-site, along with the number of participant-observers by week

Week	On-site	Off-site	Robot Users	Total Stud.	Total P-Obs.
1	4	3	3	7	6
2	3	5	4	8	5
3	7	2	1	9	5
4	5	2	2	7	6
5	5	3	2	8	6
6	6	1	1	7	6
7	4	4	1	8	7
8	4	6	3	10	6
9	5	3	1	8	3
10	5	4	1	9	4

## Appendix D Additional Information on Data Collection: Data Types and Methods

**Table 4:** Data types and methods

Method	Description	Duration	Num. of Researchers
Ethnography	46 sets of 291 pages of	10 weeks of	3–7
	thick description	2 hours teaching	
Interview	2 Classmate Interviews	30 mins.	1 (PI)
	5 Drivers Interviews	60 mins.	1 (PI)
Questionnaire	7 question digital	10 weeks. Est. 5 mins	2
	questionnaire	to complete per time.	
Participatory Design	Design workshop of	1 session of	1 (PI)
	5 participants	60 mins	

## Footnotes

1 Providing demographics specifics such as gender and race, and when combined with disability, could potentially compromise participants’ anonymity especially at our own institution. Thus, we made a joint decision between the research team and our participants, as some might seek a PhD in our field, and prefer to keep their disability characteristics confidential.
